# Comparing physical activity practice, pain and psychological characteristics in individuals with fibromyalgia and individuals with low back pain

**DOI:** 10.1186/s12998-025-00625-9

**Published:** 2026-02-05

**Authors:** Bastien Couëpel, Julien Ducas, Andrée-Anne Marchand, Marjorie Bernier, Jacques Abboud, Martin Descarreaux

**Affiliations:** 1 Department of Anatomy, Université du Québec à Trois-Rivières, 3351, boul. des Forges, C.P. 500, Trois-Rivières, QC G8Z 4M3 Canada; 2 Groupe de Recherche en Affections Neuromusculosquelettiques, 3351, boul. des Forges, C.P. 500, Trois-Rivières, QC G8Z 4M3 Canada; 3 Department of Chiropractic, Université du Québec à Trois-Rivières, 3351, boul. des Forges, C.P. 500, Trois-Rivières, QC G8Z 4M3 Canada; 4 Centre de Recherche sur l’Éducation, l’Apprentissage et la Didactique, University Brest, F-29200 Brest, France; 5 Department of Human Kinetics, Université du Québec à Trois-Rivières, 3351, boul. des Forges, C.P. 500, Trois-Rivières, QC G8Z 4M3 Canada

**Keywords:** Fibromyalgia, Low back pain, Physical activity, Pain characteristics, Psychological characteristics

## Abstract

**Background:**

Chronic primary low back pain (CLBP) and fibromyalgia (FM) have complex etiologies and management approaches. Further research is needed, particularly on subgroups like individuals with CLBP who also report signs and symptoms of FM (CLBP/FM+) to better understand their pain mechanisms and clinical profile. Examining psychological, pain-related characteristics, and physical activity could help identify subgroups of individuals with CLBP/FM+ and guide the development of targeted interventions. This study aimed to compare physical activity practice, pain and psychological characteristics of individuals with CLBP without signs and symptoms of FM (CLBP/FM−), CLBP/FM + and FM to better understand similarities and differences between these chronic pain conditions.

**Methods:**

Ninety-four individuals self-reporting CLBP (83% women) and 101 individuals self-reporting FM (96% women) were recruited online and completed a 30-min online survey. Among those with CLBP, 39 were classified as CLBP/FM+ and 55 as CLBP/FM−. Analyses of variance (ANOVAs) with polynomial contrasts were conducted to assess linear trends across the three groups on pain intensity (Numeric Rating Scale), psychological characteristics (Short Form-12, Tampa Scale for Kinesiophobia) and physical activity practice.

**Results:**

Individuals with CLBP/FM− were more likely to participate in high-intensity physical activities. The analysis revealed a significant linear trend across the CLBP/FM−, CLBP/FM+, and FM groups for all pain and psychological characteristics except for the physical component of the quality of life and moderate-intensity physical activity.

**Conclusions:**

Individuals reporting a diagnosis of FM exhibit greater symptoms severity and engage less in high-intensity physical activities than individuals with CLBP/FM+ and CLBP/FM−. Identifying a subgroup of individuals with CLBP/FM+ may help recognize mixed pain profiles and inform more personalized tailor management strategies.

## Introduction

Among the various chronic primary pain conditions, fibromyalgia (FM) and chronic primary low back pain (CLBP) are known to have complex etiologies and management [1–3], resulting in substantial personal and societal burdens [1, 4] as well as high economic costs [5, 6]. FM is defined as "chronic widespread pain accompanied by fatigue and sleep disturbances, autonomic dysfunction, cognitive impairment, hypersensitivity to external stimuli, somatic symptoms, and psychiatric disorders" [2]. CLBP is characterized as a “persistent or recurrent pain experience of more than 3 months, located between the 12th posteroinferior rib edge and the gluteal fold, that is not reliably attributed to an underlying disease process, structural lesion or deformity” [3]. FM and CLBP share abnormal pain modulation, despite having different pain distributions [7–9]. Moreover, it has been suggested that regional pain syndromes precede the development of widespread pain in most patients with FM [4]. A subgroup of patients with CLBP experiencing widespread pain following long-standing localized or regional musculoskeletal pain (CLBP/FM+) has been identified in multiple studies [7, 10–12]. These observations suggest that some individuals with CLBP may be on a continuum of chronic pain, potentially evolving toward a symptom profile similar to FM [13]. This theoretical pain continuum is defined by Nielsen and Henriksson [10] as “a continuum ranging from chronic localized/regional musculoskeletal pain to widespread chronic disabling pain”. Chronic widespread pain and FM are distinct clinical entities, as FM is generally considered more disabling and frequently associated with additional psychological symptoms [1]. Therefore, previous authors have recommended further investigation of these conditions to improve preventive and therapeutic strategies [1, 11].

Chronic pain also influences health behaviors, particularly physical activity practice, as individuals with FM or CLBP are less physically active than healthy controls [14, 15]. Physical activity recommendations in the treatment of chronic pain typically include endurance [16, 17], strength [17–20], and/or flexibility exercises [19, 20], which have positive effects on pain intensity [16, 18–20], fatigue [16, 18, 19], quality of life [19, 20], and/or stress and anxiety [17, 19] in both individuals with FM [2, 16, 18, 19, 21, 22] and CLBP [17, 20, 23, 24]. Furthermore, studies suggest that regular physical activity may slow the progression of chronic diseases [25, 26]. Despite these benefits, individuals with FM or CLBP tend to spend more time in sedentary behavior, both at work and during leisure [27, 28]. A better understanding of physical activity levels in these populations could provide valuable insights into health status, disease progression, and impact of chronic pain.

Pain-related characteristics, such as kinesiophobia and clinical pain, contribute to pain persistence in individuals with chronic pain [29, 30]. More precisely, kinesiophobia has been suggested as a major psychological barrier to physical activity [30, 31] as individuals with chronic pain, particularly those with FM or CLBP, commonly fear that movement will exacerbate their pain [32–34]. Pain is also frequently reported as a major physical barrier to exercise in qualitative studies focusing on chronic pain [30, 35], especially in CLBP [36]. These findings highlight the importance of comparing pain-related characteristics between individuals with FM and those with CLBP to better understand the impact of chronic pain on physical activity practice [15, 37, 38]. Building on a previous scoping review, relevant pain, psychological, and functional outcomes were identified across 19 studies to inform further research comparing individuals with CLBP between those with FM and to address methodological limitations observed in previous studies [39]. As individuals with CLBP/FM + were identified in this review, considering this subgroup would help clarify similarities and differences between individuals with CLBP and FM.

Therefore, the objective of this study is to compare physical activity practice, pain-related, and psychological characteristics of individuals with CLBP without signs and symptoms of FM (CLBP/FM−), CLBP/FM+ and FM to better understand similarities and differences between these chronic pain conditions. It is hypothesized that individuals with CLBP/FM− will be more physically active than individuals with FM and CLBP/FM+. It is also hypothesized that individuals with FM will demonstrate worse pain-related and psychological scores compared to the other groups.

## Methods

### Study design

This cross-sectional observational study consisted of a 30-min online survey, conducted in French on Qualtrics XM (2024) and was designed to target individuals with FM or CLBP. Participants recruitment took place between October 2022 and May 2024, with support from the *Association de Fibromyalgie* of the Quebec region and the *Association de la Fibromyalgie – Mauricie Centre-du-Québec*, as well as via social media platforms in France and Quebec. This study was approved by the Research Ethics Committee for Human Subjects (CER-22-289-07.17).

### Participants

Participants eligible for inclusion in the study were required to be between 18 and 70 years old and self-report having been diagnosed with either FM or CLBP. To be included in the FM group, participants had to self-report being primarily diagnosed with FM and meet the 2011 American College of Rheumatology (ACR) criteria [40]. For the CLBP group, participants had to self-report being primarily diagnosed with localized pain in the posterior aspect of the body from the lower part of the 12th rib to the base of the gluteal fold for more than 12 weeks [3]. To identify individuals with CLBP/FM+, participants in the CLBP group completed the 2011 ACR [40] questionnaire assessing FM-related signs and symptoms. Those presenting FM-related signs and symptoms were classified as CLBP/FM+, whereas participants without such signs or symptoms were classified as CLBP/FM−. These criteria were used to establish three groups: FM group with a self-report diagnosis of FM and unknown CLBP status (1), CLBP/FM+ group with a self-report diagnosis of CLBP and classified with FM according to the ACR questionnaire (2) and the CLBP/FM− group not classified with FM according to the ACR questionnaire (3).

Participants in all groups were excluded if they reported a prior diagnosis of any of the following conditions: inflammatory arthritis, connective tissue diseases, advanced osteoarthritis, history of spinal surgery, any neuromuscular disease, malignant tumors, uncontrolled hypertension, infection, radiculopathy, progressive neurological deficit, myelopathy, lumbar disc herniation, pregnancy, congenital heart malformation, heart disease, or metabolic diseases that could interfere with exercise.

**Fig. 1:**
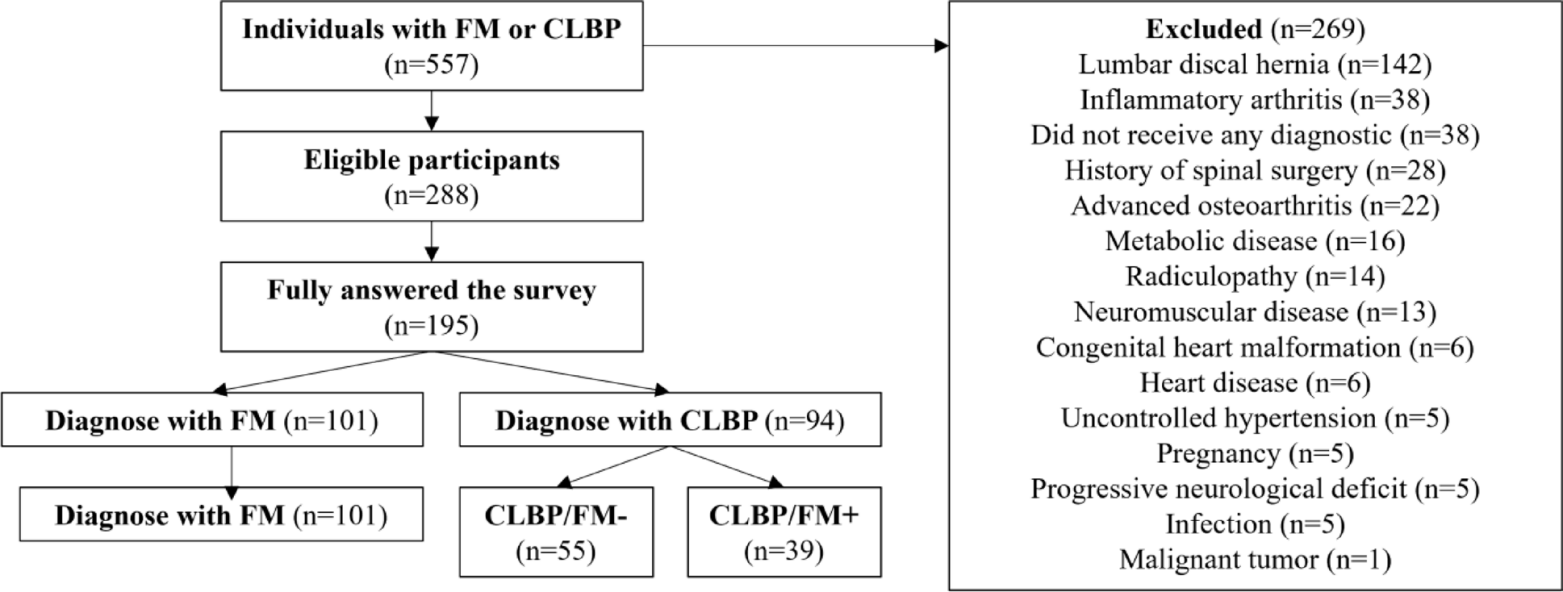
Flow diagram of the study

Figure 1 illustrates the study procedure and the number of participants at each stage. A total of 557 individuals responded to the survey (Fig. 1). Of these, 269 were excluded for meeting one or more exclusion criteria. Among the remaining 288 participants, 195 completed the 30-min survey and reported having primarily received a clinical diagnosis of FM (*n* = 101) or CLBP (*n* = 94).

### Sociodemographic outcomes

Participants were asked to provide basic anthropometric and clinical information, including their age, weight, height, and the duration of their condition. Sociodemographic data were also collected. Participants self-reported their gender identity, with options for male, female, or nonbinary. Additionally, they were asked to indicate their current socio-professional category using a predefined classification system. This system included the following categories: executives and higher intellectual professions, intermediate professions, employees, laborers, craftsmen, shopkeepers and business owners, students, retired, and non-active. These categories were adapted from the French National Institute of Statistics and Economic Studies classification of socio-professional groups [41].

### Characteristics related to fibromyalgia

A French adaptation of the 2011 questionnaire allowing the diagnosis of individuals with FM was used to measure the number of painful areas (Widespread Pain Index (WPI) score from 0 to 19) and the severity of symptoms (SS, score from 0 to 12) [40, 42]. According to this questionnaire, FM is identified by either a WPI ≥ 7 combined with an SS ≥ 5, or a WPI of 3–6 combined with an SS ≥ 9. This questionnaire was used to validate the self-report diagnosis of FM in the FM group and create the CLBP/FM + group in individuals primarily diagnosed with CLBP.

### Psychological, pain and physical activity characteristics

#### Kinesiophobia

Kinesiophobia was assessed using the French-validated version of the Tampa Scale for Kinesiophobia (TSK) [43]. This questionnaire allows for the measurement of fear of movement, or kinesiophobia, with a score out of 68. A score ≥ 37 suggests the presence of kinesiophobia [43].

#### Quality of life

Health-related quality of life was assessed using the French version of the Short Form-12 questionnaire (SF-12), a validated questionnaire measuring both physical and mental health components through 12 items. The SF-12 generates two summary scores: the Physical Component Summary (PCS) and the Mental Component Summary (MCS) [44]. Responses to the SF-12 were numerically coded and processed according to standard scoring algorithms. Each item’s response was weighted using predetermined coefficients derived from principal component analysis of the SF-36 data [45]. Higher scores indicate better perceived health for both PCS and MCS scores.

#### Intensity of pain

Participants were asked to rate their pain using a numeric rating scale from 0 (no pain) to 10 (the worst pain imaginable) through four questions: “What number best describes your general pain?“, “What number best describes your pain right now?“, “What number best describes the most intense pain you experienced in the last 24 h?“, and “What number best describes the average pain you experienced in the last 24 h?“.

#### Physical activity and sedentary behaviors

The French version of the National Observatory for Physical Activity and Sedentariness (ONAPS) was used to assess the overall physical activity level and the degree of sedentary behavior during a typical week [46]. It consists of 34 questions divided into three sections: work-related activities (A1-A7) such as time spent lifting loads or walking at work, utility-related transportation activities (B8-B19) including walking or cycling for commuting, and leisure or home-based activities (C20-C34) like exercising, gardening, or household chores. The weekly duration for moderate physical activity (MPA), vigorous physical activity (VPA) and moderate to vigorous physical activity (MVPA) is calculated using responses in hours and minutes, converted to total weekly minutes (min/week) for analysis:

$$ \begin{aligned} MPA & = (A5 \times A6) + (B9 \times B10) \\ & \quad + (B12 \times B13) + (C33 \times C34) \\ \end{aligned} $$

$$ VPA = (A2 \times A3) + (C30 \times C31) $$

$$ MVPA = MPA + VPA $$

The physical energy expenditure was estimated by multiplying minutes of each activity by its respective Metabolic Equivalent of Task (MET) value: moderate activity (4 METs), vigorous activity (8 METs), walking (3.3 METs), cycling (6 METs), and domestic tasks (2 METs), converted to total METs/min/week for analysis:

$$ \begin{aligned} MPA_{{MET}} & = 4 \times [(A5 \times A6) + (C33 \times C34)] \\ & \quad + 3.3 \times (B9 \times B10) + 6 \times (B12 \times B13) \\ \end{aligned} $$

$$ VPA_{{MET}} = {\text{ }}8 \times [(A2 \times A3) + (C30 \times C31)] $$

$$ MVPA_{{MET}} = MPA_{{MET}} + {\text{ }}VPA_{{MET}} $$

Sedentary behavior (SB) represents the daily sitting time spent at work, in transit, or at home, excluding nighttime sleep. Sedentary behavior is interpreted based on daily sitting time: < 3 h indicates low, 3–7 h moderate, and ≥ 7 h high. Total sedentary time is calculated by first converting all recorded hours and minutes into minutes, then applying the formula:

$$ SB = A7 + ~\frac{{\left( {B18~ \times ~B19} \right)~ + ~\left( {C24~ \times ~C25} \right)~ + ~\left( {C27~ \times ~C28} \right)}}{7} $$

### Sample size

Because the proportion of CLBP/FM+ individuals to be recruited could not be predetermined, the sample size was estimated based on a moderate effect size, a power of 90%, and a significance level of 0.05 (G*Power 3.1.9) for the comparison between two groups (t-test). The minimum required sample size per group was 86. However, considering potential missing data (e.g., non-responses to questions), it was estimated that 100 participants per group (200 in total) would be enough to detect significant effects.

### Statistical analysis

As the sample size in each group exceeded 30, the data distribution was assumed to be normal [47], allowing for the use of parametric tests. A first set of analyses was performed to compare individuals with FM and those with CLBP. An independent samples t-test was used to assess differences in demographic data between individuals with FM and individuals with CLBP. A chi-square test for independence was used to compare the proportion of men and women and socio-professional status among individuals with FM and individuals with CLBP.

To examine potential linear trends in pain-related, psychological characteristics, physical activity, and sedentary behaviors questionnaires' scores across three groups (FM, CLBP/FM+, CLBP/FM−) a one-way ANOVA with polynomial contrasts was conducted based on a priori hypotheses. A contrast matrix was constructed, assigning weights to each group (FM = − 1, CLBP/FM + = 0, CLBP/FM− = 1) to test for linear trends. Given the unequal sample sizes across groups, the weighted linear term was used to ensure group sizes were appropriately accounted for in the trend analysis. Prior to conducting the analysis, the homogeneity of variances was tested using Levene’s test. In cases where variances were found to be unequal (*p* < 0.05), the Welch correction was applied to account for heterogeneity. Additionally, Cohen’s d was calculated to estimate small (0.2 ≤ d < 0.49), medium (0.5 ≤ d < 0.79), and large (d ≥ 0.8) effect sizes associated with the contrasts. All analyses were conducted using IBM SPSS Statistics (Armonk, NY: IBM Corporation) for Windows version 29.0.2.0. Significance was set at *p* ≤ 0.05.

## Results

### Participants’ characteristics

Table 1 presents a comparison of sociodemographic outcomes between individuals with FM and those with CLBP. The proportions of men and women differed significantly between the two groups, as indicated by the chi-square test (χ^2^(1) = 8.590, *p* = 0.003). Additionally, individuals with FM were significantly older (t(1) = − 3.241, *p* = 0.004) than their counterparts. All other sociodemographic outcomes were not significantly different between the two groups.

**Table 1:** Sociodemographic outcomes

	Fibromyalgia (*n* = 101)	Low back pain (*n* = 94)	χ^2^ *p* value	t-test *p* value
Gender (binary)	96 W/3 M	78 W/14 M	**0.003**	–
Gender (non-binary)	2	2	0.999	–
Intermediate professions	25	33	0.107*	–
Employees	28	23		
Retired	19	14		
Students	4	7		
Craftsmen, shopkeepers, and business owners	6	6		
Executives and higher intellectual professions	0	5		
Non-active	14	4		
Laborer	4	2		
Age (years, mean ± SD)	49.36 ± 11.94	43.24 ± 14.39	–	**0.004**
BMI (kg/m^2^, mean ± SD)	29.57 ± 7.65	29.77 ± 7.39	–	0.935
Duration of condition (years, mean ± SD)	10.22 ± 8.19	10.54 ± 10.69	–	0.310

W = women; M = men; BMI = Body Mass Index; SD = standard deviation; * = the *p* value indicates that there were no significant differences in the socio-professional distribution between the two groups; bold values indicate statistically significant results (p < 0.05)

### Comparing individuals with CLBP/FM−, CLBP/FM + and FM

**Fig. 2:**
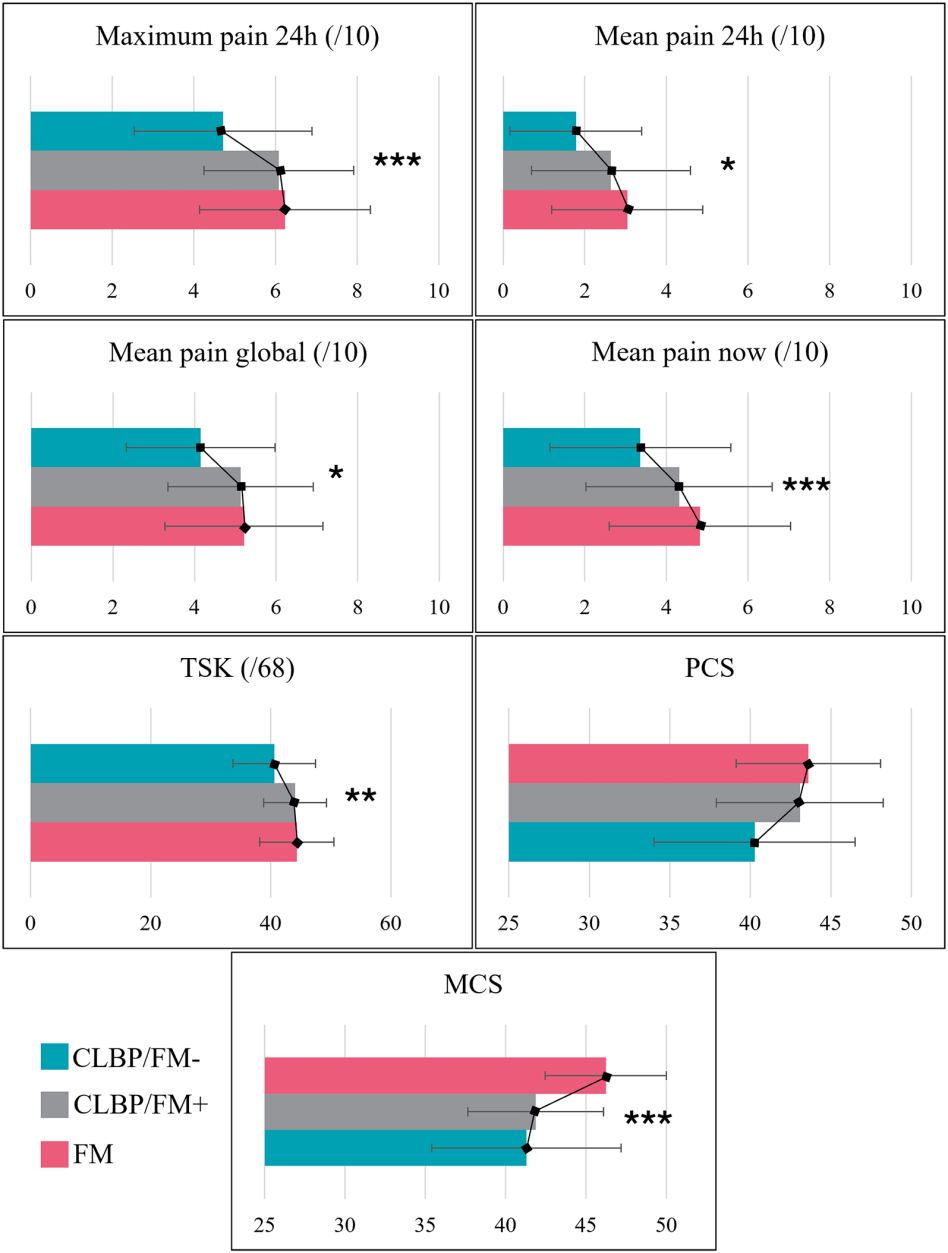
Comparison of pain-related and psychological characteristics of individuals with FM, CLBP/FM−, CLBP/FM + and FM. *Note* The mean values are presented with error bars as standard error. Asterisks indicate a significant linear trend across groups; FM = Fibromyalgia ; CLBP/FM + = Chronic Low Back Pain with signs and symptoms of fibromyalgia; CLBP/FM− = Chronic Low Back Pain without signs and symptoms of fibromyalgia; TSK = Tampa Scale for Kinesiophobia; PCS = Physical Component of the Short Form questionnaire for quality of life; MCS = Mental Component of the Short Form questionnaire for quality of life; * = *p* ≤ 0.05; ** = *p* ≤ 0.01; *** = *p* ≤ 0.001

Among the 94 individuals with CLBP who responded to the survey, 39 were identified as CLBP/FM + using the 2011 FM survey. Participants in the FM and CLBP/FM + groups presented WPI and SS scores consistent with the presence of FM (WPI = 10.93 ± 4.69 and 8.36 ± 3.29; SS = 8.52 ± 2.11 and 8.33 ± 1.71, respectively), whereas scores in the CLBP/FM − group (WPI = 3.93 ± 1.89; SS = 4.65 ± 2.18) were below the thresholds associated with FM. As presented in Table 2 and illustrated in Fig. 2, individuals with FM reported the highest scores across all pain-related and psychological measures, while individuals with CLBP/FM− had the lowest values (all *p* values < 0.05). Polynomial contrast analyses confirmed a significant linear trend for all variables (all *p* values < 0.05), except for the PCS (F(2,192) = 7.691, *p* = 0.652). Statistically significant linear increases were observed from the CLBP/FM− group to the CLBP/FM + group and then to the FM group for all pain outcomes and TSK scores. Conversely, MCS scores showed a statistically significant linear increase from the FM group to the CLBP/FM + group and then to the CLBP/FM− group. Effect sizes were the largest for the MCS (d = − 0,864 [− 1.282, − 0.443], large effect) and maximum pain (d = 0.661 [0.245, 1.076], moderate effect).

**Fig. 3:**
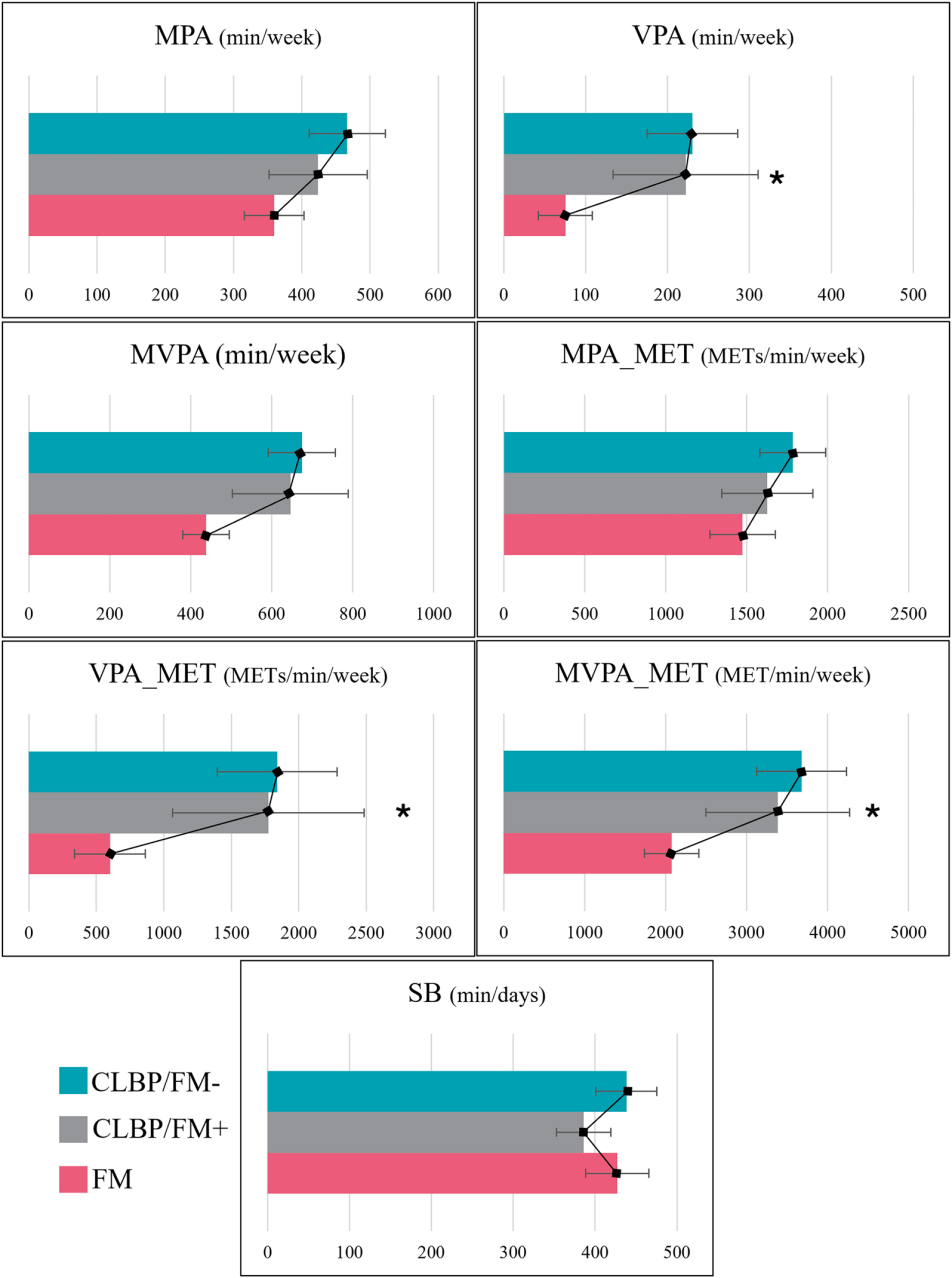
Comparison of physical activity practice and sedentary behaviors of individuals with CLBP/FM−, CLBP/FM + and FM.The mean values are presented with error bars as standard error. Asterisks indicate a significant linear trend across groups; FM = Fibromyalgia; CLBP/FM + = Chronic Low Back Pain with signs and symptoms of fibromyalgia; CLBP/FM− = Chronic Low Back Pain without signs and symptoms of fibromyalgia; MPA = Moderate Physical Activity; VPA = Vigorous Physical Activity; MVPA = Moderate to Vigorous Physical Activity; SB = Sedentary Behavior; * = *p* ≤ 0.05.

As presented in Table 3 and illustrated in Fig. 3, significant differences between groups were found for VPA, VPA_MET_ (both *p* = 0.035) and for MVPA_MET_ (*p* = 0.040). Additionally, significant linear trends were observed for VPA, VPA_MET_ (both F(2,192) = 3.407, *p* = 0.016), and MVPA (F(2,192) = 2.815, *p* = 0.026). For all these variables, time spent in physical activity increased linearly from the FM group to the CLBP/FM + group and then to the CLBP/FM− group. All other results were non-significant (*p* > 0.05).

**Table 2:** Demographic, pain-related and psychological characteristics of individuals with FM, CLBP/FM+ and CLBP/FM−

	Fibromyalgia (n = 101)	CLBP/FM+ (n = 39)	CLBP/FM− (n = 55)	*p* value ANOVA	*p* value Linear contrast test	Effect size (Cohen’s d [95% CI])
Age (years)	49.36 ± 11.94	41.72 ± 13.13	44.35 ± 15.27	**0.010**	0.375	0.201 [− 0.212, 0.613]
BMI (kg/m^2^)	29.57 ± 7.65	30.38 ± 7.24	29.32 ± 7.54	0.913	–	–
Duration of condition (years)	10.22 ± 8.19	12.64 ± 12.38	9.05 ± 9.13	0.516	–	–
Maximum pain 24h (/10)	6.23 ± 2.09	6.08 ± 1.83	4.71 ± 2.18	**< 0.001**	**<0.001**	0.661 [0.245, 1.076]
Mean pain 24h (/10)	3.04 ± 1.85	2.64 ± 1.95	1.78 ± 1.61	**< 0.001**	**0.027**	0.476 [0.062, 0.888]
Mean pain global (/10)	5.21 ± 1.94	5.13 ± 1.78	4.15 ± 1.82	**0.002**	**0.011**	0.524 [0.110, 0.937]
Mean pain now (/10)	4.82 ± 2.22	4.31 ± 2.28	3.36 ± 2.19	**< 0.001**	**0.048**	0.425 [0.012, 0.837]
TSK (/68)	44.37 ± 6.19	44.05 ± 5.23	40.58 ± 6.90	**0.001**	**0.007**	0.557 [0.143, 0.971]
WPI (/19)	10.93 ± 4.69	8.36 ± 3.29	3.93 ± 1.89	**< 0.001**	**< 0.001**	1.160 [0.732, 1.585]
SS (/12)	8.52 ± 2.11	8.33 ± 1.71	4.65 ± 2.18	**< 0.001**	**< 0.001**	1.794 [1.342, 2.241]
Physical component SF-12	40.27 ± 6.24	43.08 ± 5.18	43.61 ± 4.48	**< 0.001**	–	–
Mental component SF-12	41.29 ± 5.88	41.87 ± 4.22	46.23 ± 3.77	**< 0.001**	**< 0.001**	− 0.864 [− 1.282, − 0.443]

Results are presented as mean ± SD; CLBP/FM+ = Chronic Low Back Pain with signs and symptoms of fibromyalgia; CLBP/FM− = Chronic Low Back Pain without signs and symptoms of fibromyalgia; TSK = Tampa Scale for Kinesiophobia; WPI = Widespread Pain Index; SS = Symptom severity Scale; SF-12 = Short Form questionnaire for quality of life; bold values indicate statistically significant results (p < 0.05)

**Table 3:** Physical activity and sedentary behaviors measurement of individuals with FM, CLBP/FM+ and CLBP/FM−

	Fibromyalgia (n = 101)	CLBP/FM+ (n = 39)	CLBP/FM− (n = 55)	*p* value ANOVA	*p* value Linear contrast test	Effect size (Cohen’s d [95% CI])
MPA (min/week)	359.64 ± 429.58	423.85 ± 450.07	466.67 ± 408.13	0.321	–	–
VPA (min/week)	75.10 ± 329.33	222.05 ± 553.73	230.18 ± 411.03	**0.035**	**0.016**	− 0.382 [− 0.712, − 0.051]
MVPA (min/week)	437.84 ± 561.37	645.90 ± 892.21	674.44 ± 609.49	0.062	–	–
MPA_MET_ (METs/min/week)	1473.54 ± 1955.95	1627.54 ± 1704.19	1784.56 ± 1496.76	0.590	–	–
VPA_MET_ (METs/min/week)	600.79 ± 2634.64	1776.41 ± 4429.80	1841.45 ± 3288.25	**0.035**	**0.016**	− 0.382 [− 0.712, − 0.051]
MVPA_MET_ (METs.min/week)	2073.30 ± 3361.61	3385.72 ± 5538.31	3679.28 ± 4097.65	**0.040**	**0.015**	− 0.393 [− 0.724, − 0.062]
SB (min/days)	427.19 ± 388.39	386.06 ± 207.42	438.22 ± 276.79	0.733	–	–

Results are presented as mean ± SD ; MPA = Moderate Physical Activity ; VPA = Vigorous Physical Activity ; MVPA = Moderate to Vigorous Physical Activity ; SB = Sedentary Behavior; CLBP/FM+ = Chronic Low Back Pain with signs and symptoms of fibromyalgia; CLBP/FM− = Chronic Low Back Pain without signs and symptoms of fibromyalgia; bold values indicate statistically significant results (p < 0.05)

## Discussion

### Main results

Individuals with self-reported FM consistently exhibited more severe scores than those with CLBP/FM− and CLBP/FM + across all pain-related and psychological characteristics, including pain intensity, widespread pain, kinesiophobia, symptom severity, and quality of life. No significant differences were found between individuals with CLBP/FM−, CLBP/FM+, and FM for sedentary behaviors. Results revealed significant linear trends across all pain-related and psychological measures, except for the physical component of quality of life, with individuals with FM consistently showing the highest scores, those with CLBP/FM− the lowest, and those with CLBP/FM + demonstrating intermediate values. Similarly, linear trends indicated that time spent in vigorous and moderate-to-vigorous physical activity progressively decreased from CLBP/FM− to CLBP/FM + and FM individuals.

### Pain-related and psychological characteristics of individuals with CLBP/FM−, CLBP/FM+ and FM

The demographic characteristics (age and socio-professional status) of the FM and CLBP participants included in the present study were consistent with profiles commonly reported in the scientific literature [2, 48]. Among the 94 individuals with CLBP, 39 exhibited CLBP/FM+ characteristics based on the ACR questionnaire [40]. Interestingly, previous studies indicate that individuals with FM often report the initial onset of their condition as localized pain, frequently in the back [7, 8, 10, 49]. Participants with CLBP/FM+, initially classified as having CLBP, have been distinguished from those with CLBP/FM− by higher levels of catastrophizing, anxiety, and depression [12, 50, 51]. Moreover, Friedrich et al. [7] demonstrated a linear relationship in the severity of chronic pain profiles when studying psychological outcomes in overlapping chronic pain conditions, such as CLBP and FM. Specifically, they found that the development of severe chronic pain conditions was linearly associated with greater psychological distress, especially somatic symptoms and depression. Similarly, findings in the present study demonstrated a linear trend in pain intensity, kinesiophobia, widespread pain, symptom severity, and impaired mental quality of life across the CLBP/FM−, CLBP/FM+ and FM groups. However, this trend is unlikely to apply to all individuals with CLBP and may instead represent a trajectory for those with more severe kinesiophobia, depression and clinical pain [13]. Longitudinal studies are needed to confirm the potential evolution of chronic pain trajectories in individuals with CLBP.

While FM is generally considered a more complex and advanced condition than CLBP [7, 10], identifying potential areas of convergence in pain-related and psychological characteristics between the two conditions remains challenging [39]. Subgrouping individuals with CLBP into CLBP/FM+ and CLBP/FM− according to the ACR criteria [40] allowed for more granular comparisons between individuals with CLBP and those with FM. The presence of CLBP/FM + or other chronic pain subgroups may explain inconsistencies in previous studies on pain intensity [32, 52–56], kinesiophobia [32–34], or quality of life [52–54, 57]. Considering such subgroups could also help clarify how pain, psychological symptoms, and functional characteristics evolve over time in individuals with CLBP.

### Physical activity levels in chronic pain

As hypothesized, participants with FM were generally less physically active than those with CLBP/FM− and CLBP/FM+. Notably, CLBP/FM− and CLBP/FM+ individuals engaged more frequently in vigorous physical activity. These differences likely reflect the distinct clinical profiles of FM and CLBP, with greater symptom severity and complexity being linked to less vigorous physical activity. Larsson et al. [37] and van den Berg-Emons et al. [36] suggested that fear-avoidance beliefs and kinesiophobia may play a more critical role than pain itself in limiting physical activity. According to the fear avoidance model, high levels of pain-related fear and kinesiophobia make individuals perceive movement as threatening, maintaining physical deconditioning and emotional distress [58]. Differences between the three groups likely result from the interaction of fear-avoidance, kinesiophobia and chronic pain symptoms. Accordingly, no linear trend was observed for time spent in moderate physical activity, suggesting that such activities may be less influenced by pain and psychological characteristics associated with FM, CLBP/FM+ and CLBP/FM−. Some studies suggest a U-shaped association, with both very low and very high intensity and frequency linked to higher pain prevalence [59], while others report a linear inverse relationship, with greater activity associated with lower pain [60]. Further research is needed to clarify how physical activity interacts with psychological and clinical factors in chronic pain conditions such as FM and CLBP.

### Sedentary behaviors in chronic pain

Sedentary time did not significantly differ between individuals with FM and those with CLBP/FM− and CLBP/FM+. These findings are in line with previous data on sedentary behavior in both populations [61, 62]. Individuals with chronic pain tend to be more sedentary, and this sedentary behavior has been linked to greater pain processing impairments, regardless of physical activity levels [63]. These findings, combined with the observation that individuals with CLBP/FM− and CLBP/FM+ are more physically active than those with FM, highlight the distinction between physical activity and sedentary behaviors as separate concepts [64]. Thus, an individual with CLBP/FM− or CLBP/FM+ can be more physically active while still being as sedentary as another individual with FM. Consequently, therapeutic goals should place equal emphasis on educating patients about the risks associated with sedentary behavior alongside the promotion of physical activity in the management of chronic pain conditions [27, 65].

### Strength and limits

Physical activity levels among individuals with diverse chronic pain conditions remain understudied, although they appear essential for the optimization of the multimodal management of individuals affected by chronic pain [1]. This study addresses this gap by examining the distinct pain-related and psychological characteristics of FM, CLBP/FM− and CLBP/FM+, and how these profiles are associated with variations in physical activity levels among individuals living with these conditions.

One limitation of the present study is the reliance on self-reported physical activity levels. The use of self-reported data throughout the survey may have introduced biases, such as social desirability bias. For instance, participants may have responded based on what they believed was expected or socially acceptable, especially when reporting on health, lifestyle, or pain-related topics [66]. Another limitation of self-reported physical activity data is their subjective nature, as they rely on individual perception and recall, whereas objective physical activity data, such as those measured by accelerometers, provide more accurate and quantifiable insights into physical activity levels [14, 67]. Indeed, Schaller et al. [68] found that individuals with CLBP, as well as healthy controls, tend to overestimate their physical activity time and underestimate their sedentary behavior in self-reports compared to objective measures. Also, we were unable to confirm FM nor CLBP diagnoses through comprehensive clinical examination. Participants were included if they reported having obtained a diagnosis of FM or CLBP, which may have affected the accuracy of group classification and should be considered when interpreting the results. The use of self-reported diagnoses introduces a risk of misclassification, particularly given the known uncertainties and evolving clinical criteria on the FM diagnosis. Moreover, the diagnosis of CLBP was not assessed in participants with FM. Consequently, some participants with FM may also have had CLBP in the present sample, leading to potential overlap with the CLBP/FM+ group. It should also be noted that, although age, BMI, and socio-professional status in our sample are consistent with values reported in the scientific literature, the sample was predominantly composed of 83% women in the CLBP group and 96% in the FM group. These proportions exceed those typically reported for FM [2] and CLBP [69] and may reflect a participation bias, limiting the generalizability of the findings, particularly to men. Finally, as a priori sample size calculations were not performed, post-hoc power analyses (α = 0.05) were conducted using the observed Cohen’s d for each variable. The study was well-powered to detect effects for the main variables, such as pain variables, TSK, and MCS (power ≥ 0.858), all of which showed significant differences. In contrast, the study was underpowered for VPA and MVPA_met_ (power ≤ 0.37), so significant results for these physical activity variables should be interpreted with caution.

### Clinical applications

Variations in physical activity levels among individuals with chronic pain, in relation to their pain-related and psychological characteristics, highlight the need for personalized therapeutic programs. Such programs should aim to reduce kinesiophobia and fear-avoidance beliefs to improve engagement in physical activity, particularly vigorous activity, and to optimize associated benefits, such as improved physical function and quality of life [21, 24]. Addressing these factors may help prevent symptoms exacerbation in individuals with chronic pain. Moreover, sedentary behaviors and insufficient physical activity are distinct concepts, each associated with independent health risks [21, 24, 63]. However, given the self-reported nature of diagnoses and clinical characteristics in the present study, potential clinical applications should be interpreted with caution. Studies using validated clinical diagnoses and objective measures of physical activity are needed before further recommendations can be made.

Results of the present study also showed that individuals with CLBP/FM− and CLBP/FM+ can report engaging in higher-intensity physical activity while maintaining similar levels of sedentary behavior as individuals with FM reporting lower-intensity activity. Therapeutic programs should focus equally on promoting physical activity and reducing sedentary behaviors, even for individuals with chronic pain demonstrating high levels of physical activity.

## Conclusion

This study highlights significant differences in physical activity levels, pain-related, and psychological characteristics between individuals with CLBP/FM−, CLBP/FM+, and self-reported FM, with individuals with FM being less physically active and presenting more severe pain-related and psychological symptoms. Subgrouping individuals with CLBP allowed the identification of a CLBP/FM+ group, highlighting the relevance of this approach to help clarifying the heterogeneity of chronic pain profiles. These conclusions should be interpreted with caution, as FM status was based on self-reported diagnosis.

## Data Availability

All data generated or analysed during this study will be available upon request.
